# Interim and end‐of‐treatment PET‐CT suffers from high false‐positive rates in DLBCL: Biopsy is needed prior to treatment decisions

**DOI:** 10.1002/cam4.3867

**Published:** 2021-03-31

**Authors:** Susanna Tokola, Hanne Kuitunen, Taina Turpeenniemi‐Hujanen, Outi Kuittinen

**Affiliations:** ^1^ Department of Oncology and Medical Research Center Oulu University Hospital Oulu Finland; ^2^ Unit of Cancer and Translational Medicine Research Oulu University Oulu Finland; ^3^ Institute of Clinical Medicine Faculty of Health Medicine University of Eastern Finland Kuopio Finland; ^4^ Faculty of Health Medicine Kuopio University Hospital Kuopio Finland

## Abstract

The application of positron emission tomography (PET)‐computed tomography (CT) in treatment response evaluation has increased in diffuse large B‐cell lymphoma (DLBCL), although its predictive value is controversial. We retrospectively analyzed the rate of false‐positive PET‐CTs performed as interim (*n* = 94) and end‐of‐treatment (*n* = 8) assessments among 102 DLBCL patients treated during 2010–2017 at Oulu University Hospital. In PET‐CT Deauville score ≥4 was regarded as positive. A biopsy was performed on 35 patients, and vital lymphoma tissue was detected from nine patients. Positive biopsy findings were associated with poor disease outcomes in this study. This difference was statistically significant: 2‐year failure‐free survival (FFS) was 44% in patients with a positive biopsy versus 83% for those with a negative biopsy (*p* = 0.003). The corresponding overall survival (OS) rates were 53% versus 95% (*p* = 0.010). In the multivariate analyses, a negative biopsy was an independent protective factor in FFS (Hazard Ratio (HR) 0.093 (95% confidence interval [CI] 0.017–0.511); *p* = 0.006) unrelated to the International Prognostic Index (IPI) (HR 1.139 [95% CI 0.237–5.474] *p* = 0.871) or stage (HR 1.365 [95% CI 0.138–13.470]; *p* = 0.790). There was no statistically significant difference in OS according to the PET results, but the FFS rate was significantly higher in patients with a negative PET. The value of PET‐CT as an evaluation method suffers from a high false‐positive rate, and it is inadequate alone for the justification of treatment decisions. Biopsy results provide more reliable prognostic information for the evaluation of treatment response and outcome and should be used to assess patients with positive PET‐CT scans.

## Graphical abstract

PET‐CT is a valid method to find DLBCL patient with insufficient treatment response but suffers from high false positive rates. Biopsy is needed before treatment decisions in PET positive patients.

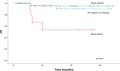

## 1 INTRODUCTION

Non‐Hodgkin lymphomas (NHL) account for 4.3% of all cancers in the USA, with DLBCL constituting 25%–50% of this number. DLBCL is the most common NHL in western countries, with an incidence of 3.81/100 000 in Europe. This incidence is increasing, and the rate of increase is higher in developed countries.^1^, ^2^, ^3^

In the past two decades, the International Prognostic Index (IPI) has been the most commonly used prognostic tool among NHL patients.^4^ Treatment outcomes from current first‐line therapies seem to be inadequate for a significant proportion of patients, despite advances and improvements. In approximately 40% of patients, the disease is primarily refractory or relapses during follow‐up.^5^, ^6^ During treatment, immunochemotherapy may provide an advantage of selection to refractory cells responsible for disease recurrence. If the disease progresses during first‐line treatment or relapses soon after, patients’ prognosis is poor, and most patients with refractory diseases have no curative treatment options.^7^, ^8^ To avoid prolonged therapies with ineffective combinations, there is a clear unmet clinical need to identify patients with primary refractory disease or insufficient treatment response at the earliest possible stage of treatment.

To improve early detection of treatment failures, PET‐CT with 18F‐fluorodeoxyglucose (FDG) as an interim (iPET) and/or end‐of‐treatment assessment has been intensively researched.^9^, ^10^, ^11^, ^12^ The role of PET‐CT in response evaluation is most indisputable as a prognostic method, with a negative predictive value of 90%–100% and a positive predictive value ranging from 50% to 82%. However, relatively good survival rates after positive PET scans reflect the challenges caused by false‐positive findings in PET‐CT.^9^, ^12^, ^13^, ^14^, ^15^

Due to the high prevalence of false‐positive rates, our aim at Oulu University Hospital has been to take a histological biopsy of all patients with PET‐positive lesions whenever possible. Patients with vital lymphoma tissue in their biopsies have been regarded as presenting a primary refractory lymphoma, implicating an intention to proceed to second‐line salvage therapy followed by autologous stem cell transplantation (ASCT) if possible. In the present study, we performed a retrospective analysis to evaluate the correlation of positive PET‐CT to the presence of vital lymphoma tissue in histological biopsy and the prognosis of patients with biopsies proving vital residual disease or a false‐positive PET‐CT.

## 2 METHODS

### 2.1 Patients, staging, and treatment

**FIGURE 1:**
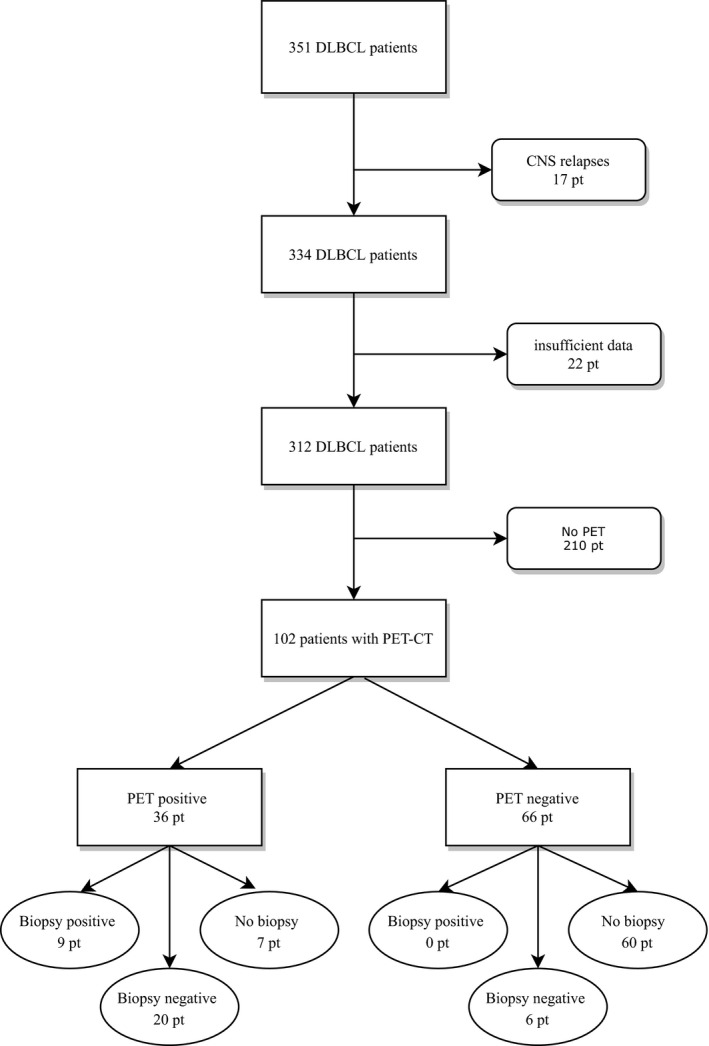
Flowchart: patient inclusion

Data were collected from Oulu University Hospital patient records, including patients treated between 1/2010 and 11/2017. The original data included 312 subjects. Seventeen patients with solitary CNS relapses and 22 patients with excessively incomplete data were excluded. From the remaining cohort (102 patients), treatment response had been evaluated by PET‐CT, and these patients were included in this analysis. (Figure 1) Within these included 102 patients, PET‐CT was performed mainly (*n* = 94) before eight cycles and was defined as interim PET‐CT. End‐of treatment PET‐CT was evaluated from eight patients and it was performed after 2 weeks from the last chemoimmunotherapy or before the first 1 month follow‐up evaluation. The need for radiotherapy was assessed by criteria defined by staging data or by interim PET‐CT if there was no option for biopsy or treatment intensification after the positive biopsy. PET‐CT scans were performed before RT treatment was started in all included cases.

As a staging examination, whole‐body CT was performed in all patients, and staging was evaluated according to the Ann Arbor classification. PET‐CT was interpreted according to the Deauville/Lugano criteria; a Deauville score of ≥4 was regarded as positive.^16^ WHO performance scores were defined between grades 0 and 4, and the IPI scale, ranging from 1 to 5, was referenced. A disease was considered primary refractory when a positive PET‐CT finding was biopsied and exhibited viable lymphoma tissue or when a radiologically verified progression occurred during treatment or within 6 months after treatment. The ethics committee of the Northern Ostrobothnia hospital district approved this study. Patient characteristics are presented in Table 1.

**TABLE 1:** Patient characteristics

			PET negative		PET positive		Biopsy positive		Biopsy negative	
Variable	Mean or No. of patients (102pt.)	SD or %	No (66pt.)	%	No (36pt.)	%	No (9pt)	%	No (26pt)	%
Gender										
Female	45	44%	33	50%	12	30%	4	44%	6	23%
Male	57	56%	33	50%	24	66%	5	56%	20	77%
Age (Years)										
<70	76	74.5%	47	71%	29	81%	9	100%	23	88%
≥70	26	25.5%	19	29%	7	19%	0	0%	3	12%
WHO Classification										
0	22	22%	16	24%	6	17%	0	0%	6	23%
1	32	31%	17	26%	15	42%	4	44%	8	31%
2	28	28%	22	33%	6	17%	2	22%	7	27%
3	7	7%	4	6%	3	8%	0	0%	3	12%
4	2	11%	1	2%	1	3%	1	11%	0	0%
No Data	11	11%	6	9%	5	14%	2	22%	2	8%
IPI										
1	7	7%	4	6%	3	8%	3	33%	1	4%
2	23	23%	11	17%	12	33%	3	33%	6	23%
3	33	32%	24	36%	9	25%	0	0%	10	38%
4	20	20%	15	23%	5	14%	2	22%	4	15%
5	7	7%	5	8%	2	6%	0	0%	3	12%
No Data	12	12%	7	11%	5	14%	0	0%	2	8%
Stage										
1	5	5%	3	5%	2	6%	1	11%	1	7%
2	13	13%	9	14%	4	11%	1	11%	3	12%
3	25	25%	16	24%	9	25%	3	33%	8	31%
4	51	50%	32	48%	19	53%	3	33%	14	54%
No Data	8	8%	6	9%	2	6%	1	11%	0	0%
B‐symptoms										
Yes	65	64%	40	61%	25	69%	5	56%	18	69%
No	29	28%	20	30%	9	25%	3	33%	8	31%
No Data	8	8%	6	9%	2	6%	1	11%	0	0%
PET Deauville Score										
1	29	28%	29	44%	0	0%	0	0%	1	7%
2	23	23%	23	35%	0	0%	0	0%	0	0%
3	14	14%	14	21%	0	0%	0	0%	5	19%
4	15	15%	0	0%	15	42%	2	22%	10	38%
5	21	21%	0	0%	21	58%	7	78%	10	38%

Patients were treated mainly with an R‐CHOP regimen (rituximab, doxorubicin, cyclophosphamide, vincristine, and prednisolone) × 6–8 (*n* = 44), R‐CHOEP (rituximab, doxorubicin, cyclophosphamide, etoposide, vincristine, and prednisolone) × 6–8 (*n* = 20), or R‐CEOP (rituximab, epirubicin, cyclophosphamide, vincristine, and prednisolone) × 4–8 (*n* = 14) with or without preface therapy. Other treatment protocols included R‐CVOP (rituximab, cyclophosphamide, etoposide, vincristine, and prednisolone) or combinations of these protocols. Patients with primary refractory or relapsed diseases were treated with salvage induction therapy with the intent to proceed to high‐dose therapy followed by ASCT. The high‐dose therapy protocols are presented in Table 2. Patients with a positive PET but a negative biopsy were treated similarly to the patients with a negative PET.

**TABLE 2:** High‐dose therapy protocols

Patient No.	First‐line	Treatment alteration after	Salvage	ASCT	Amount of CD34+cell/kg	Follow‐up status
1	R‐CHOEP x5	5th cycle	R‐MACOP‐B	1*	8.49x10E6	CR
2	R‐CHOPx8	restaging	R‐ICEx2, MACOP‐B x1, HD‐ARA‐C (ended to failure)	2		death, other reason
3	R‐CHOEPx8	restaging		2		death, disease‐related
4	R‐CHOEPx1	1th cycle	CODOX‐M‐IVAC +CODOX‐M‐IVAC	1*	4.81x10E6	death, disease‐related
5	R‐benda+R‐CHOEPx6	6th cycle	R‐DHAP+R‐ICE	1*	4.95x10E6	death, disease‐related
6	R‐CHOPx4	4th cycle	R‐DHAP+R‐ICEx2	1*	8.8x10E6	CR
7	R‐CHOPx5	5th cycle	R‐DHAPx2 + HD‐ARA‐C + R‐ICE	1*	5.25x10E6	CR
8	R‐MACOP‐B	restaging	R‐ICE +brentuximab vedotin +MAXI CHOP	1*	3.85x10E6	CR
9	R‐CEOPx8	restaging	R‐DHAPx4	1*	4.89x10E6	CR

1*, ASCT with BEAM as high dose regimen, 2, no ASCT.

The treatment response was evaluated using a CT scan and bone marrow biopsy in patients with initial bone marrow involvement; these were performed after the fourth, fifth, sixth, and/or eighth treatment cycles. PET was performed in 102 patients, most commonly after the fourth treatment cycle in 60 patients (58.8%), after the fifth in 14 patients (13.7%), and after the sixth in 15 patients (14.7%).

Follow‐up evaluation was performed at the end of treatment and every 3 months during the follow‐up for 2 years followed by every 6 months for 5 years. A response evaluation was executed by CT/PET‐CT, in accordance with the revised International Working Group response criteria, and by PET‐CT, in accordance with the Deauville/Lugano criteria.^17^, ^18^

For failure‐free survival (FFS) a disease progression, positive PET‐CT with positive biopsy, relapse of the disease, or death related to disease were considered as an event. FFS was calculated from the date of diagnosis to the date of disease relapse, disease progression, detection of primary refractory disease, disease‐related death, or the last follow‐up date, when the patient without any relapse was censored. Overall survival (OS) was calculated from the date of diagnosis to the date of death for any reason.

### 2.2 Statistics

All analyses were performed using IBM SPSS Statistics for Windows & Mac OS X. Survival analyses with corresponding *p*‐values were calculated using the Kaplan‐Meier method with the log‐rank test. Multivariate analyses were made using the Cox regression model.

## 3 RESULTS

### 3.1 Results of iPET and correlation with biopsy results

PET‐CT was performed in 102 patients as an interim evaluation (*n* = 94) and/or at the end of treatment (*n* = 8), resulting in 66 negative and 36 positive PET‐CTs. Of these 36 positive PET‐CT, 32 were interim and four were performed after the last scheduled treatment cycle. Thirty‐five patients underwent biopsies. Twenty‐six percent (*n* = 9) of the biopsies contained vital lymphoma tissue, and all these patients had positive PET‐CT. Seventy‐four percent (*n* = 26) of the biopsies were negative, and 20 of these negative biopsies were taken from patients with positive PET‐CTs. The biopsy results according to the Deauville scores are presented in Table 1.

After chemotherapy, 31 (30.4%) patients received consolidation radiotherapy (RT) due to a bulky tumor before treatment or a residual tumor after treatment. PET‐CT was performed before RT and it was positive for 10 of the 31 patients treated with consolidation RT. Biopsy was taken from eight of these 10 PET positive patient, but also from four patients with negative PET treated with RT afterward; three of these presented with vital lymphoma and nine were negative.

### 3.2 Failure‐free survival and overall survival according to iPET

Within the whole population evaluated by PET (*n* = 102), the 2‐year OS rate was 88%, and FFS was 73%, while the corresponding 5‐year rates were 85% and 63%, respectively. There were no differences in survival or progression rate based on patient age under or over 70 years.

**FIGURE 2:**
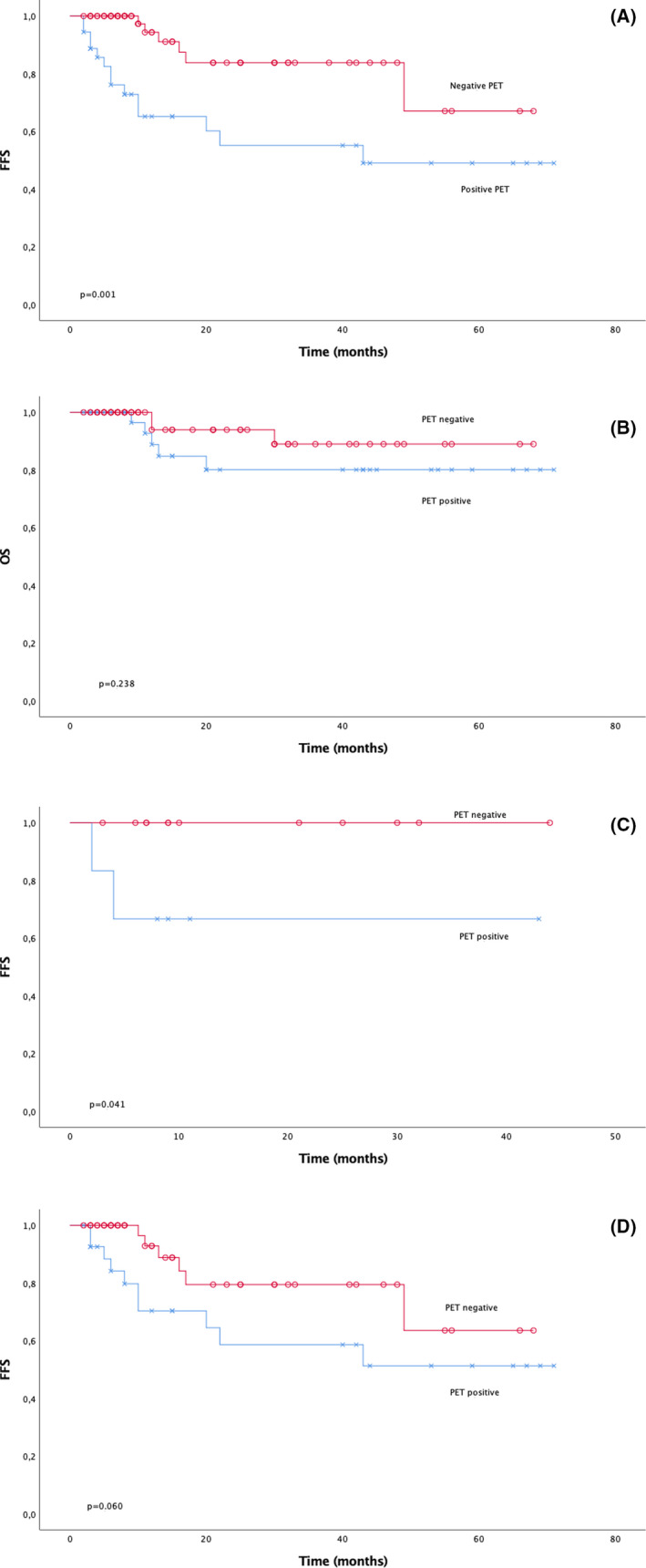
PET: (A) FFS) (B) OS (C) limited‐stage FFS (D) advanced‐stage FFS

When analyzing the data according to the PET results, FFS was more favorable in the group of PET‐negative patients, and this difference was statistically significant. The 2‐year FFS was 84% among the PET‐negative patients and 55% among the PET‐ positive patients, while the 5‐year FFS was 67% versus 49% (*p* = 0.001), respectively (Figure 2A). In the multivariate analyses, positive PET imaging was an independent risk factor for FFS (HR 3.396 [95% CI 1.189–9.701]; *p* = 0.022) unrelated to IPI (HR 0.970 [95% CI 0.291–3.238]; *p* = 0.961), stage (HR 1.136 [95% CI 0.242–7.714]; *p* = 0.723), or age (HR 0.954 [95% CI 0.263–3.458]; *p* = 0.943).

The difference in OS rates was not statistically significant, as assessed by PET: the 2‐year OS was 94% for patients with negative PET and 80% for those with positive PET. The corresponding 5‐year OS rates were 89% and 80%, respectively (*p* = 0.238) (Figure 2B).

After dividing the data into limited‐ and advanced‐stage disease, similar trends were observed. In the limited‐stage disease category, the FFS difference according to the PET results was statistically significant: both the 2‐ and 5‐year FFS rates were 100% for patients in the negative PET group versus 67% for those in the positive PET group (*p* = 0.041) (Figure 2C). There was no significant difference among patients with advanced disease, and the corresponding rates for 2‐year FFS were 80% for negative PET versus 59% for positive and, for 5‐year FFS, 64% versus 51%, respectively (*p* = 0.060) (Figure 2D).

### 3.3 Failure‐free survival and overall survival according to biopsy findings

**FIGURE 3:**
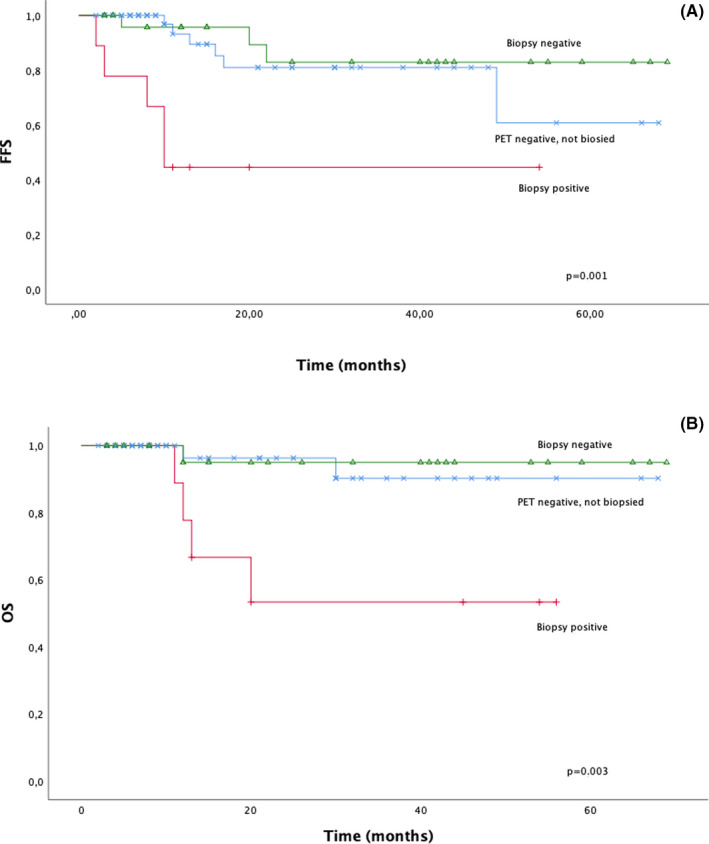
PET +biopsied and PET: (A) FFS (B) OS

When analyzed according to the biopsy findings, patients with vital lymphoma tissue in their biopsies had both 2‐year and 4‐year FFS of 44% (5 years not yet reached) versus 2‐ and 5‐year FFS rates of 83% for patients in the negative biopsy group (*p* = 0.003) (Figure 3A). In the multivariate analyses, a positive biopsy was an independent risk factor of FFS (HR 10.794 [95% CI 1.958–59.498]; *p* = 0.006) unrelated to IPI (HR 1.139 [95% CI 0.237–5.474]; *p* = 0.871) or stage (HR 1.365 [95% CI 0.138–13.470]; *p* = 0.790).

The prognostic value of the histological finding was evident in the OS rates, and the results were statistically significant. Two‐ and 4‐year OS rates were 53% in the positive biopsy patient group and 95% in the negative biopsy group (*p* = 0.010). As a reference for these OS rates, the remaining non‐biopsied patients with negative PET exhibited a 2‐year OS of 96% and a 5‐year OS of 90% (Figure 3B). The treatment and outcome details of the biopsy positive patients are presented in Table 2.

## 4 DISCUSSION

In this study, we demonstrated the high false‐positive rates of PET during and at the end of DLBCL treatment, in line with previous studies.^9^, ^10^, ^11^, ^13^ We also showed the favorable prognosis of patients with positive iPET but negative biopsies. Their FFS and OS were equal to those of patients with negative iPET. These results imply that, before modifying the therapy according to PET results, positive findings should be verified with a histological biopsy. These results also indicate the accuracy of the biopsy to help distinguish patients with truly positive PET from false positives. However, the outcomes of patients with positive biopsy specimens were poor despite intensive salvage therapies.

Almost all lymphomas are FDG‐avid, and in DLBCL the avidity is present in 97%–100% of cases.^19^ In DLBCL, FDG PET‐CT is the recommended method for staging and treatment response evaluation, including end‐of‐treatment evaluation based on the international guidelines of the National Comprehensive Cancer Network (NCCN) and the European Society of Medical Oncology. Its availability is, however, still limited, and the evaluation of response is widely based on CT.^2^, ^20^, ^21^ In primary staging, PET‐CT indicates more upstaging than downstaging compared to CT, although stage alterations have not been shown to affect treatment decisions.^13^, ^17^, ^21^, ^22^

The challenge in the evaluation of actual metabolic response using PET‐CT is its high false‐positivity rates, and all recommendations point out that treatment should be changed only if clear progression is evident.^2^, ^23^ The NCCN guidelines present PET‐CT positivity as a marker of progression while strongly recommending considering a biopsy prior to additional therapy. In cases with a negative biopsy, the recommendation is to follow the PET‐negative protocol.^21^ Most commonly, PET‐CT is assessed according to the Deauville/Lugano criteria; a Deauville score of 4 is regarded as positive and as representing active disease.^17^

Another matter of interest is whether a Deauville score of 3 should be classified as positive or negative. In the data presented here, the PET‐CT of five of the 35 biopsied patients was classified into the Deauville 3 group, and for all of them, the biopsies contained no viable tumor tissue. In the literature, the varying definition of a score of 3 is apparent in different studies, but, according to the Lugano classification, a score of 3 is assessed to present as negative and is associated with a favorable prognosis.^17^, ^19^

A meta‐analysis of a proportion of histologically verified false‐positive FDG‐PET lesions in lymphoma was published by Adams and Kwee in 2016. Six out of 13 studies included in the meta‐analysis also contained DLBCL patients. The number of biopsied patients in the studies ranged from 7 to 38, and the false‐positivity figures varied from 11.1% to 90.5%. The vast majority of the false‐positive findings were explained by inflammatory reactions. The conclusion of the meta‐analysis was that the role of interim and end‐of‐treatment FDG PET should be newly considered from a false‐positivity point of view.^15^ Our data support this conclusion.

Negative PET‐CT seems to be predictive in DLBCL according to a large meta‐analysis made by Burggraaff et al. published in 2019. This analysis included 19 studies and found a negative predictive value of 80% and the range was from 64% to 95%. This systematic review also showed the challenges in positive PET cases with the positive predictive value ranging from 20% to 74%. The conclusion was that interim FDG PET is predictive in DLBCL but the especially positive predictive value is insufficient.^24^ The prognostic value of PET‐CT compared to IPI is controversial. It seems to have some correlation with the outcome, but results are contradictory.^12^ Also, the value of end‐of‐treatment PET‐CT in lymphoma is unclear. According to the meta‐analyses published in 2019, there are no published studies that evaluate the significance of PET‐CT for improving OS compared to patients without PET‐CT evaluations.^25^ These results combined with meta‐analysis within histologically verified DLBCL patients made by Adams and Kwee in 2016, highlight the need to find those patients with false positivity in PET‐CT during the treatment as early as possible.^15^

The IPI is still the standard prognostic tool in the primary assessment of DLBCL patients. However, although PET‐CT is capable of differentiating patients into different diagnostic groups, it cannot be used to assess whether a patient has been cured. According to the current clinical treatment recommendations, salvage therapy should be initiated only after evident tumor progression. Furthermore, we must not be impervious to the poor prognosis of relapsed and refractory patients with median OS under 10 months.^7^ DLBCL is a disease entity that may promptly develop resistance to ineffective chemotherapeutic treatments. The need to identify patients with impaired response to first‐line treatment as soon as possible is paramount to maintaining possibilities for curative second‐line therapy and to spare patients from the side‐effects of ineffective treatments.

In our clinic, we try to take a histological biopsy from all patients presenting with a positive iPET‐CT. A biopsy demonstrating a viable tumor in histological examination has been considered to represent refractory disease. Among these patients, the aim has been to induce a response with salvage chemotherapy and proceed to ASCT whenever possible. In this study, we demonstrated poor FFS and significantly less favorable OS among patients with vital tumor tissues in their biopsies compared to patients with negative iPET‐CT or negative biopsies. However, the outcome in this positive biopsy group was better than after treatment intensification in a relapsed setting. All relapses occurred within 10 months, and 4‐year OS was 71% for patients treated with intensive salvage therapy. This implies that intensive salvage therapy, including high‐dose therapy, followed by ASCT might be a valid option for patients with primary refractory disease, but lack of control groups hinders the ability to make firm conclusions on this issue. From these data, we were unable to rule out the possibility that some of these patients might have been salvaged by local radiotherapy to PET‐positive sites as well.

This study was a retrospective analysis and has certain limitations. Although this dataset was large compared to previously published studies according to the number of biopsied patients, it was still limited; 35 patients underwent biopsy, and positive PET‐CT combined with a positive biopsy occurred in nine patients. Despite these limitations, we consider these findings to be reliable on the basis of the good prognosis of patients with negative biopsy specimens. These results are also in line with those previously published. To confirm these future prospective data is needed.

This study's excellent treatment results are partially explained by the exclusion of patients with CNS relapses. The biology of CNS relapses differs significantly from systemic relapses and is not reliably assessed by PET; thus, solitary CNS relapses were excluded. The risk of CNS relapse should be evaluated at the time of diagnosis, and patients at high risk should resume high‐dose methotrexate prophylaxis combined with conventional treatment.

In conclusion, according to this study, the false‐positivity rate of iPET‐CT is high, and therefore PET‐CT should only be regarded as a scanning tool for biopsies. A biopsy is needed to further stratify patients in the PET‐positive group. The prognosis of patients with positive PET‐CT and negative histology seems to be as good as the prognosis of patients with negative PET. If these results are repeatable, it should be considered whether patients with such an excellent prognosis need further follow‐up. Further studies are needed to perceive what can be done to improve the outcome of patients with a poor prognosis and whether the type of early salvage therapy that is started based on the histological residual tumor without evident radiological progression is able to improve outcome.

## CONFLICTS OF INTEREST

The authors have no conflict of interest to report.

## ETHICS STATEMENT

The ethics committee of the Northern Ostrobothnia hospital district approved this study.

## Data Availability

The data that support the findings of this study are available on request from the corresponding author. The data are not publicly available due to privacy or ethical restrictions.
